# Association between non-pharmacological therapy and healthcare use and expenditure of patients with diabetes mellitus

**DOI:** 10.1016/j.jsps.2023.06.018

**Published:** 2023-06-24

**Authors:** Abdullah Alfaifi

**Affiliations:** Department of Clinical Pharmacy, College of Pharmacy, Prince Sattam Bin Abdulaziz University, P.O. Box 173, Al-Kharj, 11942, Saudi Arabia

**Keywords:** Diabetes, Physical activity, Economic costs, Diabetes management, Diet modification

## Abstract

This study explored the sociodemographic and clinical characteristics of patients with diabetes who incorporated two non-pharmacological therapies into their lifestyle and the association between non-pharmacological therapy and healthcare utilization and expenditure. In the USA, 26.4 million people were reportedly diagnosed with diabetes and treated with diet modification or physical activity in the 2019 Medical Expenditure Panel Survey. Physical activity was defined as moderate-to-vigorous physical exercise five times per week, whereas dietary modification involved healthy eating that reduced glucose levels. Only 4.8 million patients with diabetes did not integrate any non-pharmacological intervention into their therapy regimen. Those who did not include non-pharmacological interventions had higher annual total healthcare expenditures (M = $18,428) than those who incorporated either single (M = $17,058) or dual intervention (M = $15,134). A significant difference was observed in prescribed medicine utilization per year for those who did not include lifestyle modifications or non-pharmacological interventions. Propensity score-matched participants revealed significant differences in hospital stays, outpatient visits, and emergency department expenditures. Patients with diabetes who adhered to two non-pharmacological interventions showed significantly lower healthcare utilization. Being active and following a healthy diet can help prevent the progression of diabetes mellitus complications and reduce the cost associated with diabetes.

## 1 Introduction

Diabetes mellitus (DM) is a group of metabolic diseases characterized by complex and chronic hyperglycemia, requiring continuous management beyond only pharmacological interventions (ElSayed et al., 2023). According to the International Diabetes Federation, approximately 537 million people worldwide have been diagnosed with DM (International Diabetes Federation, 2021). In addition, the Centers for Disease Control and Prevention (2022) reported that 37.3 million Americans live with DM. The estimated cost of diabetes in the United States is approximately $294.6 billion, including direct medical costs and reduced productivity (Williams et al., 2020). Specifically, care for patients with diabetes accounts for 1 in 4 healthcare dollars in the United States because of the high medical expenditures associated with the disease. Moreover, indirect costs must also be considered, including those related to lower productivity at work or an inability to work, along with lost productivity from deaths attributed to diabetes (American Diabetes Association, 2018).

Physical activity and dietary changes are the cornerstones of lifestyle modifications designed to prevent and manage DM and related morbidities (American Diabetes Association Professional Practice Committee, 2022). The ideal management of DM mostly focuses on controlling glucose levels within the normal physiologically recommended range characterized by a healthy subject and minimizing the risk of hypoglycemia (Raveendran et al., 2018). Physical activity, particularly cardiorespiratory fitness, is the primary approach for reducing diabetes incidence and its impact. Some researchers have reported that strength training can also prevent diabetes and enable patients to slowly reduce their medication use (Colberg et al., 2016). Similarly, Thomas and Elliott (2010) contended that physical activity in the form of aerobic exercises, such as swimming, yoga, and continuous pattern movement, could help prevent morbidity and mortality in patients with DM. The researchers’ findings revealed an exercise-related reduction of 39–70% and 50% in the prevalence of type 2 and type 1 DM, respectively. Furthermore, a trial intervention involving resistance exercises demonstrated improved glycemic control levels and decreased insulin resistance in patients with type 2 diabetes.

Maiorino et al. (2017) described a healthy diet as following a healthy eating plan that strictly controls a patient’s consumption of fat, sugar, carbohydrates, and alcohol and emphasizes the consumption of vegetables, fruits, whole grains, and fat-free or low-fat dairy products. In a study examining the relationship between food intake and blood glucose levels, Thomas and Elliott (2010) discovered that type 2 diabetes was associated with obesity. Additionally, the authors discussed the glycemic index, which ranks foods based on their effect on blood glucose levels, with a diet with heavy carbohydrate intake having the highest effect on blood glucose levels. Based on these observations, improving patients’ diets and reducing their weight may lead to better diabetic outcomes. Currently, there are no studies that compare the effect of different lifestyle modifications on the use of healthcare services and related expenditures. This study aimed to explore the healthcare use and costs associated with lifestyle modifications in patients with DM. A sub-analysis comparison was conducted to explore which non-pharmacological therapy has significantly higher association in reducing healthcare use and expenditure. The study also investigated the distribution of sociodemographic and clinical characteristics based on the number of non-pharmacological interventions incorporated.

## 2 Materials and methods

We performed a retrospective observational database analysis of data from the Medical Expenditure Panel Survey (MEPS). The MEPS consists of a set of large-scale surveys of families, individuals, medical providers, and employers across the United States (https://meps.ahrq.gov/mepsweb). The independent variable was a non-pharmacological intervention. The groups consisted of those who followed dual non-pharmacological interventions, only one intervention, and no non-pharmacological intervention. Dual non-pharmacological interventions included diet modification and physical activity. In this study, physical activity was defined as moderate-to-vigorous physical exercise at least five times per week. Diet modification was defined as healthy eating that resulted in reduced glucose levels or diet changes to manage and treat diabetes.

It is important to note that these two questions were part of the Diabetes Care Survey (DCS) administered by MEPS. DCS is a self-administered paper-and-pencil questionnaire. Patients received a DCS based on their response to whether they were ever told by a doctor or health professional that they had diabetes. Diet modification question was (Is your diabetes being treated by modifying your diet?). Physically active patients with DM in the study sample were identified through their responses to the survey question, “Do you presently spend 30 min or more in moderate to vigorous physical activity at least five times a week?”

Other independent variables were considered before assessing the association between the two non-pharmacological interventions and healthcare utilization and expenditure to avoid overestimation or underestimation of the true intervention effect. These variables included age, sex, race/ethnicity, educational attainment, marital status, family income, insurance status, presence of comorbid conditions, and diabetes duration.

The outcome variables were healthcare use and expenditure. The collection of healthcare utilization information relied solely on the MEPS household component, which organized the data assembled in each survey into five categories: office-based, hospital outpatient department, hospital emergency department, hospital inpatient department, and prescribed medicine. Utilization data were collected at the event level for each participant. As a result, these variables largely denoted the full year of use, and the data they provided could be used to calculate the yearly utilization data for 2019.

The MEPS defines expenditure as the sum of direct payments for healthcare services during the survey year, including out-of-pocket payments and payments through private or public insurance. The medical care component of the MEPS collected, imputed, and validated data from a sample of office-based visits to physicians, ambulatory events, and prescribed medicines. The major expenditure categories included total office-based, outpatient departments, emergency facilities, hospital inpatients, and prescription medication expenditures. The five categories of expenditure variables and other categories, such as dental expenditures, formed an aggregate total healthcare expenditure variable.

A series of chi-square tests were conducted to investigate the sociodemographic and clinical differences between the groups. A generalized linear model of a gamma distribution with the expenditure category as the dependent variable and the reported non-pharmacological mode as the main independent variable was used. For the utilization variables, a Tweedie distribution was used. This model has an advantage over other models because it overcomes the issue of data with multiple zeros. Independent variables were added to the model to estimate the true intervention effects.

A subanalysis was conducted to explore the differences between the two modes of non-pharmacological intervention using propensity score matching (PSM). The central role of PSM in observational studies is to more accurately determine causal effects, as selection bias occurs most commonly in the estimation of causal effects in observational studies and arises from a lack of resemblance between the individuals being studied (Rosenbaum and Rubin, 1983, Imai et al., 2008). Adjustment based on PSM reduces selection bias and confounding, which are the two most important concerns regarding internal validity in observational studies (Little and Rubin, 2000). Therefore, in this study, this method required the formation of matched sets of patients with diabetes who only exercised and those who only followed diet modifications with similar propensity scores. The selection from the pool of comparison patients was made from only those with similar demographic and clinical characteristics. This study utilized 1:1 greedy matching, which is the most commonly used method for PSM (Parsons, 2001). All analyses relied on MEPS sample clustering, stratification, and weight adjustments using the R (v3.5.3) studio-integrated development environment.

## 3 Results

**Fig. 1:**
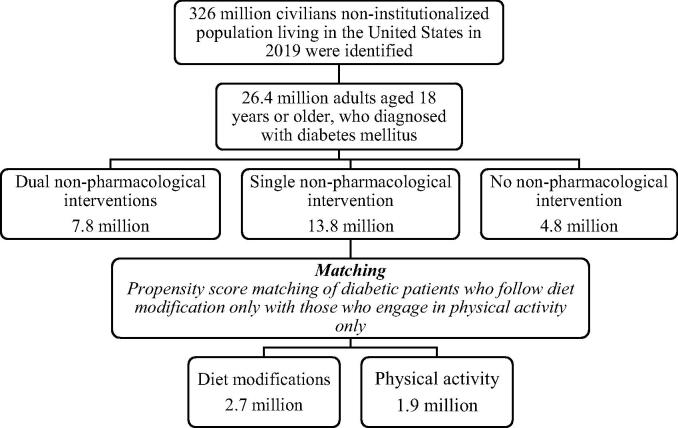
Flowchart of patients with diabetes mellitus in the MEPS. The flowchart illustrates the number of diabetic patients diagnosed and categorized into three major groups: patients including single, dual, and no mode of non-pharmaceutical interventions. The patients utilizing a single non-pharmaceutical intervention were further subanalyzed to determine differences in the two modes of non-pharmaceutical intervention.

In total, 26.4 million adults aged ≥ 18 years reported being diagnosed with diabetes mellitus in the USA. The descriptive findings showed that approximately 81% of the patients with DM used at least one mode of non-pharmacological intervention (Fig. 1). Overall, a higher percentage of patients with diabetes used both modes of non-pharmacological interventions, and female patients were more likely to use a single mode. Patients identified as Caucasian in the race/ethnicity questionnaire predominated in all groups, whereas Hispanics accounted for 16.4% and 17.8% of the dual and single intervention groups, respectively. The sociodemographic and clinical characteristics of the patients are presented in Table 1.

**Table 1:** Descriptive statistics of patients with diabetes in medical expenditure panel survey.

Variables	Dual, %	Single, %	None, %	*p*-value ^a^
Weighted Frequency	7,790,811	13,775,265	4,807,413	
**Age category**				0.901
18–39	7.4	8.2	6.2	
40–64	46.4	46.3	46.4	
65+	46.2	45.5	47.4	
**Sex**				0.0005
Male	57.5	46.4	52.5	
Female	42.5	53.6	47.5	
**Race/ethnicity**				0.2903
Hispanic	16.4	17.8	11.9	
White	58.1	59.6	62.2	
African American	16.9	13.5	16.5	
Other or multiple races	8.6	9.1	9.4	
**Marital status**				0.4014
Married	53.5	54.0	49.8	
Previously married ^b^	32.4	31.4	38.1	
Never married	14.1	14.6	12.1	
**Education level**				0.0461
<High school	33.0	38.9	32.4	
High school diploma	51.3	44.5	53.2	
College or higher	15.7	16.6	14.4	
**Insurance**				0.2913
Insured	96.8	96.4	98.3	
Uninsured	3.2	3.6	1.7	
**Body mass index**				0.0798
Underweight	7.2	9.8	6.6	
Normal	13.5	11.5	13.6	
Preobese	32.1	26.2	23.6	
Obese	47.2	52.5	56.2	
**Income category**				0.1812
Poor or low income	33.1	36.8	40.0	
Middle income	31.5	28.1	31.8	
High income	35.4	35.1	28.2	
**Charlson Comorbidity Index**				0.4773
0	63.7	60.2	58.2	
1	21.6	22.3	22.5	
≥2	14.7	17.5	19.3	
**Diabetes duration;** mean (SE)	12.7 (0.55)	13.7 (0.43)	15.5 (1.1)	<0.0001

^a^ p-values were found using chi-square tests for differences from those with dual, single, and no non-pharmacological intervention for categorical variables and Taylor series method for continuous variable.

^b^ Previously married includes widowed, divorced, and separated individuals.

The proportion of patients with a higher socioeconomic status seemed to be higher among those following at least a single mode of non-pharmacological intervention compared to those with poor or near-poor economic status. The differences in the percentage of family income between the groups were not statistically significant. Most patients with DM were educated beyond high school. A chi-square test of independence was conducted by comparing the types of lifestyle modifications by the three levels of education completed by the MEPS respondents, and a significant interaction was found.

The healthcare utilization according to the use of non-pharmacological interventions is shown in Table 2. Patients with diabetes who engaged in physical activity and diet modification tended to use office-based care and were prescribed fewer medicines than their counterparts. When the difference between the total number of inpatient visits was analyzed, the results revealed no significant differences between the three groups. However, a significant difference in outpatient department utilization was observed between the various non-pharmacological intervention groups. Furthermore, patients who adhered to healthy lifestyles used emergency room services less often. The generalized linear model test revealed no significant differences between the groups.

**Table 2:** Mean annual healthcare utilization by non-pharmacological intervention groups.

	**Dual**		**Single**		**None**		
**Sample size (n)**	591		1,030		347		
**Population size (N)**	7,790,811		13,775,265		4,807,413		
Utilization categories	Mean	95% CI	Mean	95% CI	Mean	95% CI	*p*-value ^a^
Prescribed medicine (numbers)	33.5	30.8–36.3	39.7	37.4–42.2	45.8	40.5–51.2	<0.0001
Hospital stays (days)	0.68	0.40–0.97	1.11	0.80–1.4	1.76	0.72–2.79	0.0007
Inpatients visits (numbers)	0.20	0.14–0.27	0.24	0.19–0.29	0.27	0.18–0.37	0.0842
Outpatient visits (numbers)	1.2	0.84–1.5	1.7	1.3–2.1	1.5	0.68–2.2	0.0003
Office-based visits (numbers)	10.7	9.3–12.2	13.5	12.2–14.8	13.1	10.1–16.1	0.0064
Emergency room visits (numbers)	0.36	0.27–0.45	0.42	0.37–0.48	0.46	0.34–0.57	0.2294

^a^ p-values were found using a generalized linear model using a Tweedie distribution for differences among those using dual, single, and no non-pharmacological intervention.

A generalized linear model was used to compare the mean total office-based expenditures of patients with diabetes. Patients who did not participate in any lifestyle modification were found to have greater average expenditures than those with dual or single modes of non-pharmacological intervention. The differences in total outpatient facility expenditures between the groups were calculated, and the results showed a significant difference in expenditures among the three groups. In addition, patients who used dual interventions exhibited greater average hospital inpatient expenditures than those without or with a single intervention. Analysis of emergency room expenditures revealed a significant difference among the three groups. Patients with no intervention had significantly higher prescribed medication expenditures than their counterparts. As expected, the sum of all healthcare expenditures of the patients with no intervention was significantly higher than that of the patients with single or dual interventions. The healthcare expenditures according to the use of non-pharmacological interventions are shown in Table 3.

**Table 3:** Mean annual healthcare expenditures according to the non-pharmacological intervention group.

	Dual		Single		None		
**Sample size (n)**	591		1,030		347		
**Population size (N)**	7,790,811		13,775,265		4,807,413		
Expenditures categories	Mean	95% CI	Mean	95% CI	Mean	95% CI	*p*-value ^a^
Prescribed medicine ($)	4,945	4,222–5,668	6,293	5,750–6,835	7,076	5,894–8,259	0.0149
Hospital stays ($)	4,499	1,623–7,375	3,290	2,315–4,265	4,319	1,833–6,806	<0.0001
Outpatient cost ($)	844	586–1,101	1,524	9,16–2,133	1,055	439–1,671	0.0277
Office-based cost ($)	3,012	2,160–3,865	3,376	2,718–4,033	3,052	2,452–3,652	0.0534
Emergency room cost ($)	308	213–402	383	310–456	412	247–578	<0.0001
Total Healthcare cost ($)	15,134	11,803– 18,464	17,058	15,234–18,881	18,428	14,606– 22,250	0.0006

^a^ p-values were found using a generalized linear model using a gamma distribution for differences among those with dual, single, and no non-pharmacological intervention.

The annual healthcare utilization between the physical activity only and diet only groups is presented in Table 4. The subanalysis revealed that patients who incorporated physical activity as the only lifestyle modification had significantly fewer prescribed medicines and office-based visits than those who relied solely on diet modifications. However, the expenditures associated with these utilization categories differ. The cost of prescribed medicines was lower in the physical activity group, while the cost of office-based visits was lower for those who only used diet modification. Finally, hospital stay and outpatient costs were significantly lower among patients who managed their diabetes with a healthy diet (Table 5).

**Table 4:** Mean annual healthcare utilization between physical activity only and diet only groups among propensity-scored matched subjects.

	Physical activity group		Diet modification group		
Matched sample (n)	163		163		
Population size (N)	1,898,897		2,365,079		
Utilization categories	Mean	95% CI	Mean	95% CI	*p*-value ^a^
Prescribed medicine (numbers)	33.5	29.4–37.2	39.6	35.2–44.1	0.0028
Hospital stays (days)	1.4	0.8–1.9	1.1	0.3–2.0	0.6387
Inpatients visits (numbers)	0.2	0.1–0.4	0.4	0.1–0.3	0.1644
Outpatient visits (numbers)	1.2	0.7–1.7	1.0	0.7–1.3	0.2365
Office-based visits (numbers)	9.0	7.5–10.5	13.6	11.2–16	<0.0001
Emergency room visits(numbers)	0.32	0.23–0.41	0.51	0.37–0.66	0.1567

^a^ p-values were found using generalized linear models with a Tweedie distribution for differences between groups.

**Table 5:** Mean annual healthcare expenditures between physical activity only and diet only groups among propensity-scored matched subjects.

	Physical activity group		Diet modification group		
Matched sample (n)	163		163		
Population size (N)	1,898,897		2,365,079		
Utilization categories	Mean	95% CI	Mean	95% CI	*p*-value ^a^
Prescribed medicine ($)	5,388.8	4,714.8–7,012.8	5863.8	4,392.2–6,385.5	0.0496
Hospital stays ($)	7,035.1	3,598.4–10,471.9	2,690.6	1,210.6–4,170.6	0.0109
Outpatient cost ($)	1,122.4	444.3–1,800.3	760.3	562.9–957.6	<0.0001
Office-based cost ($)	3913.1	3343.2–4482.8	4204.4	2784.0–5624.8	0.0085
Emergency room cost ($)	326.8	220.7–432.8	468.3	206.9–729.6	0.0219
Total healthcare cost ($)	18,922	14562.1–23281.5	16,040	13395.6–18684.7	0.0003

^a^ p-values were found using generalized linear models with a gamma distribution for differences between groups.

## 4 Discussion

At the time of this study, the number of patients with DM was 26.4 million, among whom 19% were physically inactive and did not follow a healthy diet. This finding revealed that a small number of patients with diabetes did not follow the American Diabetes Association recommendations for managing diabetes. However, a higher prevalence of physical inactivity has been reported in other studies. For example, a study conducted in Taiwan revealed that 51.9% of participants were physically inactive, 35.0% were insufficiently active, and 13.1% were sufficiently active (Su et al., 2020). The present study found that dual modes of non-pharmacological intervention were more prevalent among men than women, which is consistent with the findings of previous studies (Morrato et al., 2007). This disparity should be addressed by increasing awareness of the importance of non-pharmacological interventions among female patients with diabetes.

Our results showed that patients using single or no lifestyle modifications had a significantly higher number of office-based visits and prescribed medicines than those using dual interventions. Moreover, those using dual non-pharmacological interventions had lower costs in every expenditure category. Physical inactivity and unhealthy diets have adverse effects on health and are directly linked to high medical expenditure and healthcare utilization (Bueno et al., 2016, Gebreslassie et al., 2020). Therefore, it is not surprising that patients who were physically active and consumed a healthy diet had lower healthcare utilization.

The subanalysis showed a significant difference between those who were physically active and followed no dietary moderation and those who modified their diet but did not engage in physical activity. The total direct cost for patients engaging in physical activity only was estimated to be $2,882 greater than that for patients solely relying upon diet modification. The estimates from this study show that relying on physical activity alone may produce a much greater economic burden. These average cost estimates underscore the importance of both modes. However, in terms of prescribed medicines and office-based visits, diet modifications were more effective in reducing patients both healthcare use and costs.

One of the most notable findings of this study is that expenditures outcomes were significantly higher among those who did not incorporate lifestyle modifications compared to their counterparts in all the classifications of healthcare services, whereas some of the healthcare utilization classifications (hospital stays, emergency department services, and outpatient department stays) were not significantly different between the three groups. Previous studies have documented that patients with diabetes have fewer interactions with the healthcare structure (Brewster et al., 2020). This may clarify the discrepancy in healthcare utilization and expenditures, thus accounting for the lack of statistical significance in healthcare utilization. However, the results also showed that when patients had access to medical care, they incurred higher costs than patients who engaged in physical activity and diet modifications, which in turn led to substantial healthcare expenditures.

Although this study contributes to the existing literature, it has several limitations. While the MEPS database provides valuable information, it has several drawbacks, as most of the study variables were self-reported by the patients. This disadvantage was mitigated using diagnoses provided by the MEPS medical provider component to validate the self-reported diagnoses. The second limitation of using the MEPS database is the lack of information regarding the nature of diet modification. Diet modification in the DCS questionnaire is to show if the patient is treating his/her diabetes with diet. It does not reveal the type of diabetes meal planning or medical nutrition therapy utilized. Moreover, MEPS lacks information regarding diabetes type, diabetes severity, hemoglobin A1C tests, and type of sports activity used by the patients. Thus, the results of this study should be interpreted carefully.

Nevertheless, the impact of the present study could be extended to future research on the specific components of healthcare services available to patients with diabetes, such as over-the-counter medication and healthy diet and gym membership costs. Future studies should be conducted using a large-scale, nationally representative sample of patients with diabetes to identify missing costs and services.

## 5 Conclusion

The results of this study indicate that non-pharmacological interventions can be effective in managing diabetes and reducing healthcare utilization. Patients who engaged in single or dual modes of non-pharmacological intervention had significantly lower healthcare expenditures and use than those who did not participate in lifestyle modifications. This finding is consistent with previous research demonstrating the cost-effectiveness of lifestyle interventions in managing diabetes and reducing healthcare costs. Diet modification in patients with DM produces better healthcare cost and utilization estimates than relying solely on physical activity. Non-pharmacological interventions can play an important role in managing diabetes and reducing healthcare expenditure. Healthcare providers should consider incorporating lifestyle interventions into treatment plans for patients with diabetes to improve patient outcomes and reduce healthcare utilization, especially in female patients with diabetes.

## Institutional review board statement

The MEPS 2019 data and authorization forms follow the Health Insurance Portability and Accountability Act (HIPAA). The Westat Institutional Review Board under a multi-project assurance (MPA M−1531) reviewed and approved the HIPAA compliance of the MEPS forms. established. The confidentiality of the MEPS data is addressed in Sect. 924(c) of the Act.

## Informed consent statement

All participants provided informed consent prior to their participation in the MEPS. All experimental protocols were approved by Westat, Inc and Research Triangle Institute under the authority of the Public Health Research Act.

## Funding

This study is supported via funding from Prince Sattam bin Abdulaziz University project number (PSAU/2023/R/1444).

## CRediT authorship contribution statement

**Abdullah Alfaifi:** Conceptualization, Methodology, Software, Validation, Formal analysis, Investigation, Resources, Data curation, Writing – original draft, Writing – review & editing.

## Declaration of Competing Interest

The authors declare that they have no known competing financial interests or personal relationships that could have appeared to influence the work reported in this paper.
